# Regulation of protumorigenic pathways by Insulin like growth factor binding protein2 and its association along with β-catenin in breast cancer lymph node metastasis

**DOI:** 10.1186/1476-4598-12-63

**Published:** 2013-06-16

**Authors:** Priyanka Sehgal, Neeraj Kumar, Varuvar Rajesh Praveen Kumar, Shilpa Patil, Animesh Bhattacharya, Manavalan Vijaya Kumar, Geetashree Mukherjee, Paturu Kondaiah

**Affiliations:** 1 Department of Molecular Reproduction, Development and Genetics, Indian Institute of Science, Bangalore 560012, India; 2 Departments of Pathology, KMIO, Bangalore, India; 3 Department of Surgery, KMIO, Bangalore, India

**Keywords:** IGFBP2, Breast cancer, Wnt signaling, β-catenin, Integrin

## Abstract

**Background:**

Insulin like growth factor binding proteins modulate the mitogenic and pro survival effects of IGF. Elevated expression of IGFBP2 is associated with progression of tumors that include prostate, ovarian, glioma among others. Though implicated in the progression of breast cancer, the molecular mechanisms involved in IGFBP2 actions are not well defined. This study investigates the molecular targets and biological pathways targeted by IGFBP2 in breast cancer.

**Methods:**

Transcriptome analysis of breast tumor cells (BT474) with stable knockdown of IGFBP2 and breast tumors having differential expression of IGFBP2 by immunohistochemistry was performed using microarray. Differential gene expression was established using R-Bioconductor package. For validation, gene expression was determined by qPCR. Inhibitors of IGF1R and integrin pathway were utilized to study the mechanism of regulation of β-catenin. Immunohistochemical and immunocytochemical staining was performed on breast tumors and experimental cells, respectively for β-catenin and IGFBP2 expression.

**Results:**

Knockdown of IGFBP2 resulted in differential expression of 2067 up regulated and 2002 down regulated genes in breast cancer cells. Down regulated genes principally belong to cell cycle, DNA replication, repair, p53 signaling, oxidative phosphorylation, Wnt signaling. Whole genome expression analysis of breast tumors with or without IGFBP2 expression indicated changes in genes belonging to Focal adhesion, Map kinase and Wnt signaling pathways. Interestingly, IGFBP2 knockdown clones showed reduced expression of β- catenin compared to control cells which was restored upon IGFBP2 re-expression. The regulation of β-catenin by IGFBP2 was found to be IGF1R and integrin pathway dependent. Furthermore, IGFBP2 and β-catenin are co-ordinately overexpressed in breast tumors and correlate with lymph node metastasis.

**Conclusion:**

This study highlights regulation of β-catenin by IGFBP2 in breast cancer cells and most importantly, combined expression of IGFBP2 and β-catenin is associated with lymph node metastasis of breast tumors.

## Background

The Insulin like Growth Factor binding proteins (IGFBP) are a family of six proteins that bind with high affinity to Insulin like growth factors (IGF-I and IGF-II), prolong their half-life in circulation and thereby regulate IGF actions. Insulin like growth factor binding protein 2 (IGFBP2) is the second most abundant IGFBP in circulation and in a context dependent manner it can either inhibit or potentiate the actions of IGF [1], thereby modulating the prosurvival and/or mitogenic effects of IGF. Elevated expression of IGFBP2 has been observed in multiple malignancies, including Glioblastoma multiforme [2-4], ovarian [5,6], pancreatic [7], gastric [8], prostate [9], colon [10], breast [11,12], leukemia [13] and thyroid cancer [14]. In addition, increased expression of IGFBP2 has been correlated with poor prognosis in prostate, glioblastoma and colon cancers [15-18]. It has been reported that IGFBP2 inhibits the IGF dependent proliferation of normal cells while in tumor cells, it promotes proliferation in an IGF1R dependent or independent manner [19,20]. Pro proliferative action of IGFBP2 has been reported in prostate, ovarian and colon cancer cells and non-transformed rat osteoblasts [19,21-24]. IGFBP2 expression has also been shown to enhance migration and invasion in glioma, ovarian and bladder cancer cells [3,25-27]. Recent studies in glioma implicate IGFBP2 in the activation of PI3K Akt pathway [28], integrin/ILK/NF-B network which drives glioma progression in mice [29] and binding to integrin α5 [30] that brings about increased migration and invasion. In breast cancer, IGFBP2 over expression has been shown to confer drug resistance [11] and increased expression has been reported to correlate with lymph node metastasis In T1 breast carcinomas [31]. However, mechanisms that govern IGFBP2 actions in breast cancers are poorly understood.

In the present study, to elucidate the cellular pathways influenced by IGFBP2 in breast cancer, gene expression profiling of IGFBP2 knockdown breast cancer cells was compared with the expression profile of IGFBP2 positive breast tumors. Our results highlight regulation of cell cycle and Wnt signaling pathways by IGFBP2. Most significantly, our data shows for the first time that the concomitant over expression of IGFBP2 and β-catenin in breast cancer is associated with increased incidence of lymph node metastasis.

## Results

### IGFBP2 perturbation by shRNA alters gene expression profile in breast cancer cells

**Figure 1:**
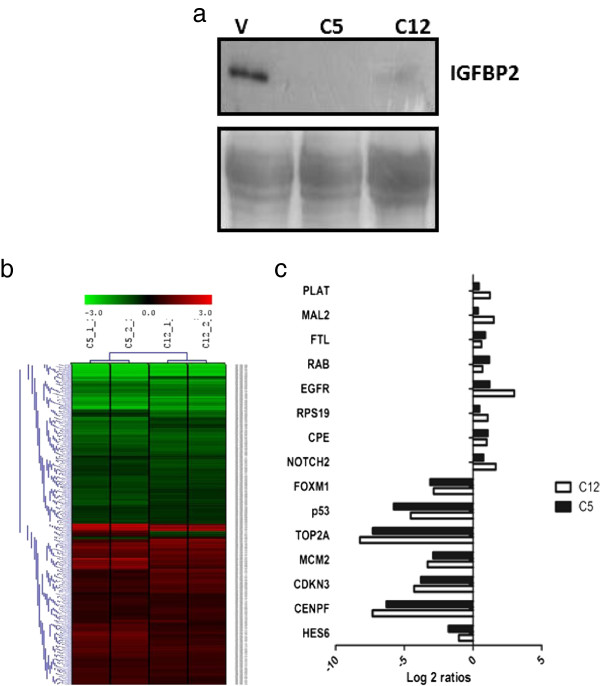
**IGFBP2 regulated genes in BT474 breast cancer cells. a**) Western blot analysis of IGFBP2 in the supernatant of IGFBP2 knockdown clones C5 and C12 and control cells. Lower panel is ponceau stained membrane shown as loading control. **b**) Hierarchical cluster of differentially expressed genes in IGFBP2 knockdown BT474 cells versus control cells. Differentially regulated genes were clustered using MeV software. The dendrogram on the left shows different clusters of genes segregated according to the pattern of regulation. Red and green indicate high and low expression of genes respectively. Black indicates no regulation. **c**) Validation of selected genes by qPCR. Bar graphs of differentially regulated genes in IGFBP2 Knockdown BT474 clones versus control cells. The graphs represent the fold change over control after normalization with the expression of RPL35a.

In view of the pro-tumorigenic actions of IGFBP2 reported in several cancers including breast tumors, we decided to delineate the molecular mechanism of IGFBP2 actions in breast cancers. Initially, stable sub lines of breast tumor cell line BT474 with knockdown of IGFBP2 were generated. Among several clones, two of the clones (C5 and C12) that showed considerable knock down of IGFBP2 (Figure 1a) were selected for further studies. Transcriptome analysis of the IGFBP2 knock down cells using Agilent whole human genome 4x44K arrays was performed against control cells (vector transfected). Data analysis revealed significant regulation of 4069 probes in both the clones compared to control cells. Among these, 2067 probes showed up regulation while 2002 probes showed down regulation (Additional file 1: Table S1). Hierarchical cluster revealed similar expression pattern of regulated genes in both the clones (Figure 1b). The list of top 25 up and down regulated genes is shown in Table 1. The differentially regulated genes were subjected to pathway enrichment analysis using GSEA (Table 2). This analysis revealed enrichment of down regulated genes belonging to cell cycle, DNA replication, repair, p53 signaling, oxidative phosphorylation, Wnt signaling, etc. qPCR analysis of some genes validated differential expression seen in microarray data (Figure 1c). Over expression of IGFBP2 in the knockdown cells resulted in up regulation of IGF1R, IGF2, TOP2A, p53, CCND1 and FOXM1 genes which were down regulated upon IGFBP2 knockdown (Additional file 2: Figure S1) suggesting the specificity of the regulation of these genes by IGFBP2. Hence, perturbation of IGFBP2 results in differential expression of several genes and pathways.

**Table 1:** List of top 50 differentially regulated genes (p < 0.05) in IGFBP2 knockdown clones

**Probe**	**Gene symbol**	**Accession no.**	**LOG2 ratios IGFBP2 (shRNA/scrambled)**	
			**C5**	**C12**
A_23_P70007	HMMR	NM_012484	−6.1994	−6.3340
A_23_P115872	CEP55	NM_018131	−5.5493	−5.7555
A_23_P155815	HCAP-G	NM_022346	−5.9516	−5.6434
A_23_P49878	FAM64A	NM_019013	−5.3231	−5.5775
A_23_P401	CENPF	NM_016343	−5.6859	−5.5456
A_24_P297539	UBE2C	NM_181803	−6.0374	−5.5064
A_23_P52017	ASPM	NM_018136	−5.6626	−5.4146
A_23_P118815	BIRC5	NM_001012271	−5.7127	−5.3579
A_23_P51085	SPBC25	NM_020675	−5.3333	−5.2314
A_32_P62997	PBK	NM_018492	−5.3592	−5.1651
A_23_P379614	OIP5	NM_007280	−5.4136	−5.0805
A_23_P138507	CDC2	NM_001786	−5.3918	−4.9418
A_23_P74349	CDCA1	NM_145697	−5.0628	−4.7854
A_23_P375	CDCA8	NM_018101	−4.9988	−4.7055
A_23_P385861	CDCA2	NM_152562	−4.8413	−4.6523
A_23_P107421	TK1	NM_003258	−5.1628	−4.6138
A_23_P50108	KNTC2	NM_006101	−4.5643	−4.6060
A_23_P124417	BUB1	NM_004336	−4.8189	−4.5874
A_23_P88331	DLG7	NM_014750	−4.8666	−4.5641
A_23_P118834	TOP2A	NM_001067	−4.9585	−4.5030
A_24_P234196	RRM2	NM_001034	−4.6703	−4.4466
A_23_P65757	CCNB2	NM_004701	−4.6828	−4.4265
A_23_P119943	IGFBP2	NM_000597	−2.4765	−4.4074
A_23_P88731	RAD51	NM_002875	−4.9625	−4.3833
A_23_P133123	MND1	NM_032117	−4.6389	−4.3051
A_23_P31407	AGR2	NM_006408	4.8060	4.2255
A_23_P106194	FOS	NM_005252	2.1989	4.2063
A_23_P429998	FOSB	NM_006732	1.5980	3.8822
A_23_P113952	AY227436	AY227436	3.4910	3.8165
A_23_P500000	SCEL	NM_144777	3.6586	3.2728
A_23_P169437	LCN2	NM_005564	2.5385	3.2159
A_23_P380754	PRSS1	NM_002769	2.3278	3.1664
A_23_P29773	LAMP3	NM_014398	4.2963	3.1041
A_23_P5983	PLTP	NM_006227	1.3270	2.8955
A_23_P310274	PRSS2	NM_002770	2.0382	2.8886
A_23_P71170	TRPV6	NM_018646	2.6719	2.7426
A_23_P98121	FXYD4	NM_173160	3.0264	2.6979
A_23_P88095	TBC1D4	NM_014832	0.9759	2.6576
A_32_P230828	GAS5	NR_002578	1.9990	2.6280
A_23_P369343	KLK8	NM_144505	2.0683	2.5972
A_23_P18684	CLGN	NM_004362	2.4448	2.5689
A_32_P37592	SCARNA17	NR_003003	1.7724	2.5574
A_23_P22735	BEX2	NM_032621	3.3099	2.5138
A_24_P921446	EMP1	BC017854	1.1796	2.4896
A_23_P121926	SEPP1	NM_005410	2.7851	2.4722
A_23_P34915	ATF3	NM_004024	3.5213	2.4288
A_23_P105803	FGF9	NM_002010	0.9259	2.3751
A_24_P327886	TCEA3	NM_003196	2.1369	2.3522
A_23_P391344	RASGEF1A	NM_145313	3.5570	2.3442
A_24_P18190	HSPA5	NM_005347	2.8335	2.3293

**Table 2:** GSEA summary of pathways associated with genes down regulated upon IGFBP2 knockdown

**NAME**	**SIZE**	**NOM p-val**	**FDR q-val**	**FWER p-val**
KEGG_CELL_CYCLE	49	0	0	0
KEGG_DNA_REPLICATION	28	0	0	0
KEGG_NUCLEOTIDE_EXCISION_REPAIR	15	0	0	0
KEGG_MISMATCH_REPAIR	15	0	0	0
KEGG_PATHOGENIC_ESCHERICHIA_COLI_INFECTION	15	0	2.19E-04	0.001
KEGG_HOMOLOGOUS_RECOMBINATION	15	0	1.83E-04	0.001
KEGG_PYRIMIDINE_METABOLISM	21	0	1.56E-04	0.001
KEGG_OXIDATIVE_PHOSPHORYLATION	19	0	0.00635	0.039
KEGG_PARKINSONS_DISEASE	21	0	0.01229	0.082
KEGG_PROGESTERONE_MEDIATED_OOCYTE_MATURATION	17	0.006186	0.013514	0.098
KEGG_SPLICEOSOME	32	0.002012	0.01617	0.128
KEGG_OOCYTE_MEIOSIS	30	0	0.02404	0.199
KEGG_P53_SIGNALING_PATHWAY	26	0	0.027815	0.247
KEGG_HUNTINGTONS_DISEASE	30	0.004098	0.032137	0.3
KEGG_WNT_SIGNALING_PATHWAY	21	0.045726	0.138961	0.804
KEGG_PURINE_METABOLISM	26	0.047431	0.150692	0.844
KEGG_GAP_JUNCTION	17	0.15251	0.155502	0.877
KEGG_TGF_BETA_SIGNALING_PATHWAY	15	0.393214	0.318836	0.995
KEGG_SMALL_CELL_LUNG_CANCER	15	0.55144	0.400925	0.999
KEGG_REGULATION_OF_ACTIN_CYTOSKELETON	25	0.517578	0.687552	1
KEGG_TIGHT_JUNCTION	16	0.787018	0.687305	1
KEGG_PATHWAYS_IN_CANCER	52	0.229814	0.731651	1
KEGG_ALZHEIMERS_DISEASE	38	0.433663	0.706543	1

*NOM P* nominal P value.

### Differential expression of genes between tumors staining positive or negative for IGFBP2

**Figure 2:**
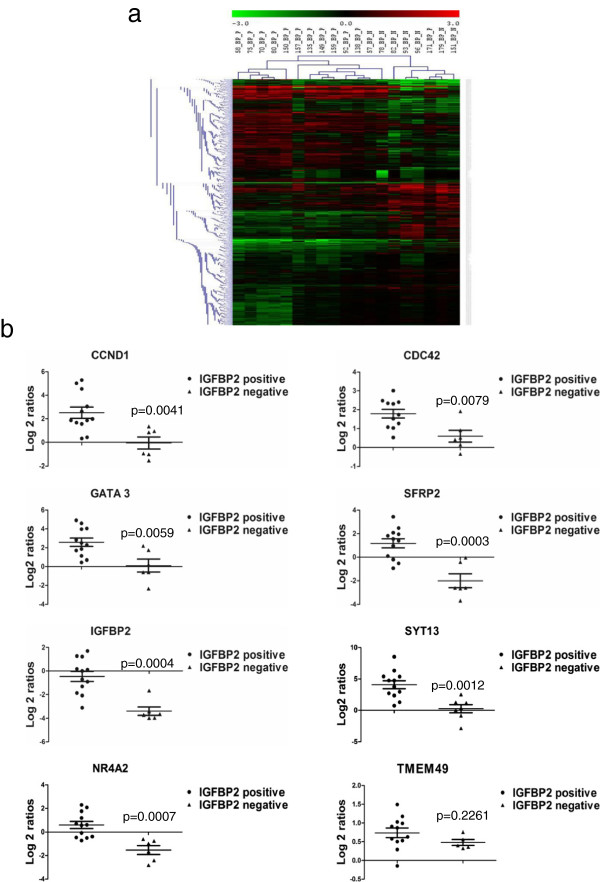
**Differential expression of genes in IGFBP2 positive and IGFBP2 negative tumor samples compared to control tissues. a**) Genes were clustered using MeV software. The dendrogram on the left shows different clusters of genes segregated according to the pattern of regulation. Red and green indicate high and low expression of genes respectively. Black indicates no regulation. **b**) Validation of selected genes. Scatter plots of differentially regulated genes in IGFBP2 positive and IGFBP2 negative tumors compared to the expression in normal tissues. Log 2-transformed gene expression ratios obtained from real-time quantitative PCR analysis normalized to TBP expression are plotted. Each dot represents a data derived from one sample.

In order to determine, whether expression of IGFBP2 regulated genes as revealed by IGFBP2 perturbation is also altered in tumors, we studied the gene expression patterns in tumors based on IGFBP2 expression. We selected 12 IGFBP2 positive and 7 IGFBP2 negative tumor RNAs for microarray expression analysis using Agilent whole human genome 4x44K arrays. Comparison of gene expression profiles between IGFBP2 positive and negative tumors revealed 3460 probes as significantly differentially regulated. Among them, 1635 probes were up regulated and 1825 probes were found to be down regulated in IGFBP2 positive tumors compared to IGFBP2 negative tumors (Additional file 3: Table S2). List of top 25 up or down regulated genes are shown in Table 3. To identify enriched pathways associated with differentially expressed genes, Gene set enrichment analysis (GSEA) was carried out. The genes up regulated in IGFBP2 positive tumor samples showed significant enrichment in Focal adhesion, MAPK signaling pathway, apoptosis, Chemokine signaling, cytokine-cytokine receptor interaction and ECM receptor interaction and Wnt signaling pathway (Table 4). Hierarchical cluster (Euclidean distance method) of log2 transformed differentially expressed genes between IGFBP2 positive and negative tumors revealed two major clusters consisting of predominantly either IGFBP2 positive or negative tumors. However, in one cluster, there is a sub cluster representing exclusively IGFBP2 positive tumors (Figure 2a). Microarray results were validated on few genes by qPCR. As shown in Figure 2b, qPCR revealed that CCND1(Cyclin D1), CDC42, GATA 3, SYT13 and SFRP2 and TMEM49 as up regulated in IGFBP2 positive tumors while IGFBP2, NR4A2 and SFRP2 were down regulated in IGFBP2 negative tumors. In addition, since Wnt pathway genes were significantly regulated in IGFBP2 knock down cells, we studied the expression of Wnt target genes in IGFBP2 positive and negative breast tumors. The Wnt target genes CCND1, SFRP2 (Figure 2b) MCAM, SP5 and IGF1 (Additional file 4: Figure S2) were found to be differentially expressed between IGFBP2 positive and negative tumors. Taken together, the data from the IGFBP2 knockdown cells and IGFBP2 positive breast tumors suggest a positive correlation of IGFBP2 with pro-tumorigenic pathways including Wnt pathway in breast cancer.

**Table 3:** List of top 50 differentially regulated genes (p < 0.05) in IGFBP2 positive versus IGFBP2 negative tumors

**Probe**	**Gene name**	**Accession no.**	**Fold change IGFBP2 +/ IGFBP2-**
A_23_P161940	SCGB2A2	NM_002411	4.2566
A_23_P8702	PIP	NM_002652	2.8894
A_24_P137501	SFRP2	NM_003013	2.4919
A_23_P312300	SCGB2A1	NM_002407	2.4797
A_24_P347431	FOXA1	NM_004496	2.4462
A_23_P372234	CA12	NM_001218	2.3044
A_23_P393099	TFF3	NM_003226	2.2590
A_23_P215328	SFRP4	NM_003014	2.2567
A_23_P213745	CXCL14	NM_004887	2.1824
A_23_P413641	PREX1	NM_020820	2.1719
A_23_P99063	LUM	NM_002345	2.1563
A_23_P75056	GATA3	NM_001002295	2.0939
A_24_P322771	TFF1	NM_003225	2.0888
A_23_P119943	IGFBP2	NM_000597	2.0290
A_23_P161659	SYT13	NM_020826	1.9984
A_23_P329768	GREB1	NM_014668	1.9528
A_23_P105212	THRSP	NM_003251	1.9163
A_23_P95594	NAT1	NM_000662	1.9107
A_24_P264943	COMP	NM_000095	1.8625
A_23_P89431	CCL2	NM_002982	1.8472
A_32_P133072	SPON1	NM_006108	1.8374
A_23_P33196	COL5A2	NM_000393	1.8357
A_23_P2920	SERPINA3	NM_001085	1.8217
A_23_P22970	PIK3R3	NM_003629	1.7963
A_23_P165778	MLPH	NM_024101	1.7915
A_32_P184464	ROPN1	NM_017578	−3.5588
A_23_P328545	GABRP	NM_014211	−2.9846
A_23_P66137	SOX8	NM_014587	−2.7293
A_24_P417407	ROPN1B	NM_001012337	−2.5309
A_23_P53176	FOLR1	NM_016725	−2.3657
A_23_P56197	CRLF1	NM_004750	−2.2530
A_23_P369343	KLK8	NM_144505	−2.2248
A_24_P236251	DLK1	NM_003836	−2.1531
A_23_P78248	KRT23	NM_015515	−2.1320
A_23_P47484	GLYATL2	NM_145016	−2.1208
A_23_P10127	SFRP1	NM_003012	−2.0505
A_23_P40108	COL9A3	NM_001853	−2.0500
A_23_P47616	FOLH1	NM_004476	−1.8461
A_23_P50815	TTYH1	NM_020659	−1.7817
A_23_P137173	TMSL8	NM_021992	−1.7054
A_23_P110234	CSN1S1	NM_001890	−1.6859
A_23_P216448	NFIB	NM_005596	−1.6243
A_23_P78980	B3GNT3	NM_014256	−1.5946
A_24_P924484	K03200	K03200	−1.5836
A_32_P157391	PSMAL	NM_153696	−1.4833
A_23_P110837	IRX4	NM_016358	−1.4820
A_23_P43157	MYBL1	X66087	−1.4800
A_23_P59960	CRISPLD1	NM_031461	−1.4630
A_23_P422212	SLC35F3	NM_173508	−1.4618
A_23_P37205	NDRG2	NM_201535	−1.4531

**Table 4:** GSEA summary of pathways associated with genes up regulated in IGFBP2 positive tumors

**NAME**	**SIZE**	**NOM p-val**	**FDR q-val**	**FWER p-val**
KEGG_MAPK_SIGNALING_PATHWAY	43	0	0	0
KEGG_LEISHMANIA_INFECTION	20	0	0	0
KEGG_CYTOKINE_CYTOKINE_RECEPTOR_INTERACTION	35	0	0.007717	0.013
KEGG_SYSTEMIC_LUPUS_ERYTHEMATOSUS	19	0.001976	0.006687	0.015
KEGG_T_CELL_RECEPTOR_SIGNALING_PATHWAY	19	0.00211	0.016915	0.045
KEGG_FOCAL_ADHESION	30	0.007619	0.025984	0.084
KEGG_COMPLEMENT_AND_COAGULATION_CASCADES	16	0.015968	0.023016	0.087
KEGG_ECM_RECEPTOR_INTERACTION	15	0.002053	0.022288	0.096
KEGG_CHEMOKINE_SIGNALING_PATHWAY	32	0.008403	0.020502	0.1
KEGG_APOPTOSIS	20	0.008584	0.018452	0.1
KEGG_VIRAL_MYOCARDITIS	17	0.005906	0.017723	0.105
KEGG_NATURAL_KILLER_CELL_MEDIATED_CYTOTOXICITY	27	0.028902	0.037213	0.228
KEGG_GNRH_SIGNALING_PATHWAY	19	0.044444	0.113272	0.563
KEGG_CELL_ADHESION_MOLECULES_CAMS	26	0.095808	0.195608	0.805
KEGG_ALZHEIMERS_DISEASE	24	0.087302	0.183226	0.805
KEGG_PATHWAYS_IN_CANCER	48	0.098	0.185527	0.831
KEGG_AXON_GUIDANCE	17	0.11553	0.176978	0.834
KEGG_NEUROACTIVE_LIGAND_RECEPTOR_INTERACTION	24	0.136538	0.210733	0.893
KEGG_DILATED_CARDIOMYOPATHY	15	0.165339	0.237117	0.925
KEGG_NEUROTROPHIN_SIGNALING_PATHWAY	19	0.168932	0.240447	0.932
KEGG_MELANOGENESIS	15	0.252033	0.367091	0.99
KEGG_LYSOSOME	18	0.329389	0.436596	0.996
KEGG_CALCIUM_SIGNALING_PATHWAY	19	0.367886	0.427407	0.996
KEGG_WNT_SIGNALING_PATHWAY	24	0.361616	0.414545	0.996
KEGG_ENDOCYTOSIS	20	0.365503	0.423908	0.998
KEGG_ANTIGEN_PROCESSING_AND_PRESENTATION	15	0.419958	0.454951	0.999
KEGG_HUNTINGTONS_DISEASE	18	0.568465	0.60777	1
KEGG_REGULATION_OF_ACTIN_CYTOSKELETON	29	0.74269	0.769736	1
KEGG_PURINE_METABOLISM	20	0.933054	0.931545	1

*NOM P* nominal P value.

### Common genes differentially expressed in breast tumors and cell lines based on IGFBP2 expression

In the previous experiments, we identified genes differentially expressed in breast tumors and breast cancer cells lines based on IGFBP2 expression. In order to identify the genes commonly regulated by IGFBP2 in cell lines and tumors, we compared the gene expression profiles of IGFBP2 positive versus negative tumors and IGFBP2 knockdown breast cancer cells. 654 probes were found to be common among IGFBP2 regulated genes in tumors and cell line. Among these 412 probes were down regulated in IGFBP2 positive tumors and up regulated upon IGFBP2 knockdown while 242 probes were up regulated in IGFBP2 positive tumors and down regulated upon IGFBP2 knockdown (Additional file 5: Table S3). Some genes that are differentially regulated in both are shown in Table 5. Genes such as FBLN1, ID1, FN1, LMO2, DCK, TLR4 which have important roles in tumor progression were up regulated in IGFBP2 positive tumors and were decreased upon IGFBP2 knockdown in breast cancer cells whereas genes such as SRPRB, POPDC3, ARHGEF4, KCNN4, BC11A which have negative role in tumorigenesis were down regulated in IGFBP2 positive tumors and were up regulated in IGFBP2 negative cells (p < 0.05). These results indicate that these genes or the pathways associated with these genes could be truly regulated by IGFBP2 in breast cancer. Some of these genes/pathways may have a role in IGFBP2 mediated tumor progression.

**Table 5:** List of top 50 common genes differentially regulated between IGFBP2 positive tumors and IGFBP2 knockdown clones

**Probe**	**Gene symbol**	**Accession no.**
A_23_P119943	IGFBP2	NM_000597
A_23_P118392	RASD1	NM_016084
A_23_P211631	FBLN1	NM_006486
A_23_P53126	LMO2	NM_005574
A_23_P22433	RP2	NM_006915
A_23_P252306	ID1	NM_002165
A_24_P119745	FN1	NM_212482
A_23_P70307	SMOC2	NM_022138
A_23_P170986	TMCC1	NM_001017395
A_32_P140139	F13A1	NM_000129
A_23_P204850	RB1	NM_000321
A_32_P216004	KCTD9	AF130091
A_23_P83939	SYAP1	NM_032796
A_23_P259438	DCK	NM_000788
A_32_P28284	TPM4	NM_003290
A_23_P42257	IER3	NM_003897
A_32_P152348	HIST1H2BD	BQ683489
A_32_P66881	TLR4	NM_138554
A_32_P89709	TPM1	NM_001018004
A_23_P380848	TXNL5	NM_032731
A_24_P106297	AMACR	NM_014324
A_23_P401055	SOX2	NM_003106
A_23_P89799	ACAA2	NM_006111
A_23_P500799	CASP6	NM_001226
A_23_P134237	RARRES2	NM_002889
A_23_P113952	AY227436	AY227436
A_23_P88095	TBC1D4	NM_014832
A_23_P369343	KLK8	NM_144505
A_23_P371039	NTSR1	NM_002531
A_24_P844917	AF222023	AF222023
A_23_P90601	STEAP3	NM_182915
A_24_P57047	DLL3	NM_203486
A_23_P363426	SRP46	NM_032102
A_23_P204751	ACCN2	NM_020039
A_23_P11262	F8A1	NM_012151
A_23_P376591	CLYBL	NM_206808
A_23_P80773	SRPRB	NM_021203
A_23_P253221	ARHGEF4	NM_032995
A_24_P163237	STOX2	NM_020225
A_23_P8240	FAM50B	NM_012135
A_23_P67529	KCNN4	NM_002250
A_24_P411186	BCL11A	NM_022893
A_23_P358597	POPDC3	NM_022361
A_23_P78518	CEACAM19	AK128234
A_23_P91702	EIF3S7	NM_003753
A_23_P415558	ZNF212	NM_012256
A_24_P414269	ALG3	NM_005787
A_23_P130764	KCNJ14	NM_170720
A_24_P152635	TXNDC14	NM_015959
A_23_P82478	PUS7	NM_019042

KEGG pathway analysis of common differentially regulated genes between IGFBP2 perturbed cells and IGFBP2 positive tumors revealed that the regulated genes belong to Glioma, Oxidative Phosphorylation, Apoptosis, Pathways in cancer and ErbB signaling pathway (Additional file 6: Table S4).Taken together, these data indicate that tumors with IGFBP2 expression phenotype are associated with distinct changes in expression of genes associated with the regulation of cell proliferation and tumorigenicity.

### β-catenin expression is regulated by IGFBP2 in breast cancer cells

**Figure 3:**
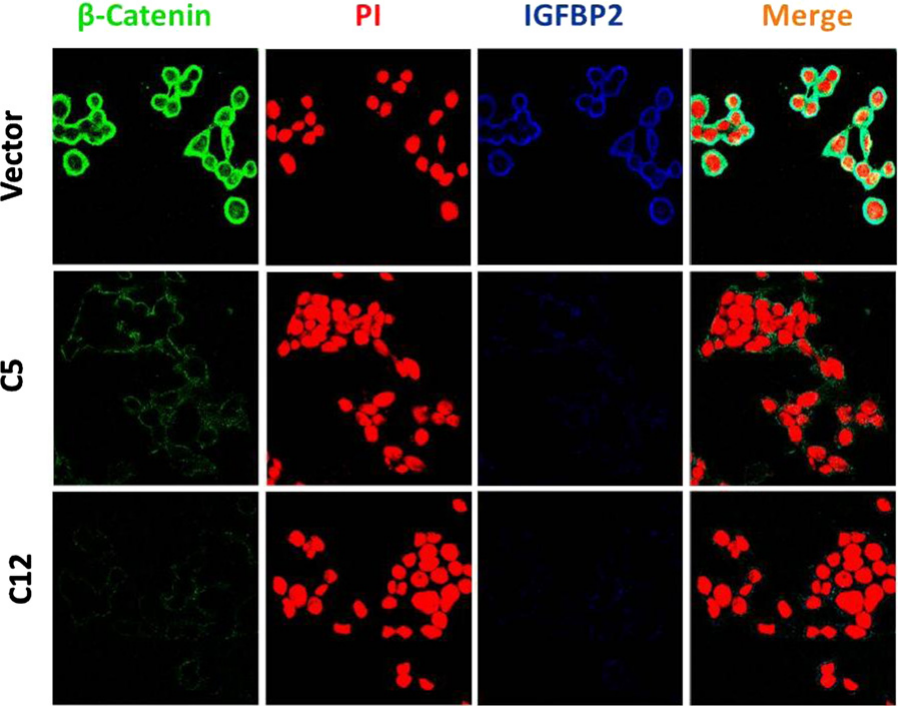
**β-catenin and IGFBP2 expression in IGFBP2 knockdown and control cells.** IGFBP2 knockdown clones and control cells were plated on coverslips and allowed to grow. 24 h after plating, cells were fixed, permeabilized, and stained for β-catenin and IGFBP2 expression. Expression of β-catenin and IGFBP2 is shown in green and blue, respectively. Nucleus was stained using propidium iodide (PI) as shown in red. Original magnification was 63×.

**Figure 4:**
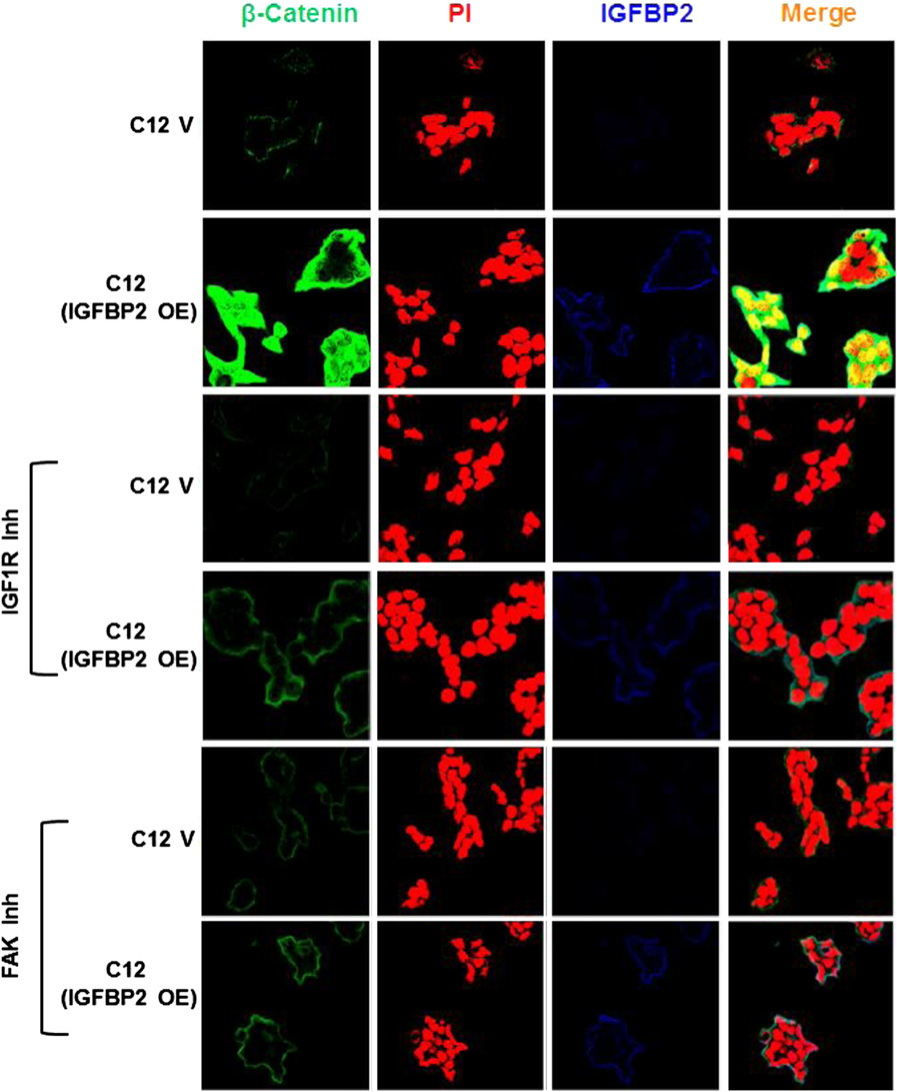
**Regulation of β-catenin by IGFBP2 is IGF1R and FAK dependent.** IGFBP2 knockdown clone C12 was transfected with IGFBP2 and 36 h. after transfection cells were fixed and analyzed for β-catenin, and IGFBP2. For inhibitor experiments, 24 h. after transfection cells were treated with IGF1R or FAK inhibitor for 12 h. Cells were fixed and analyzed for β-catenin and IGFBP2 expression. Expression of β-catenin and IGFBP2 is shown in green and blue, respectively. Nucleus was stained using propidium iodide (PI) as shown in red. Original magnification was 63×. V, Vector control; OE, Over expression; Inh, inhibitor.

Since the GSEA analysis of differentially expressed genes in both tumors and knockdown cells revealed significant regulation of Wnt signaling pathway, we decided to examine if IGFBP2 regulates Wnt pathway. As β-catenin is an effector of Wnt pathway we determined β-catenin expression in IGFBP2 knockdown cells. As shown in Figure 3, knockdown of IGFBP2 in BT474 breast cancer cells substantially decreased the expression of β-catenin in both the clones C5 and C12, suggesting a direct regulation of β-catenin by IGFBP2. In good correlation, when IGFBP2 expression is restored in the knockdown cells, β-catenin expression is also restored (Figure 4). These results collectively demonstrate regulation of β-catenin expression by IGFBP2.

It has been known that some of the IGFBP2 actions are mediated in part by the activation of IGF1 receptor and also through integrin receptors [20]. Hence, in order to identify the intermediates of IGFBP2 regulation of β-catenin, we studied the effect of IGF1R inhibitor (PPP, 10 μM) and Focal Adhesion Kinase inhibitor (PP2, 10 μM) on the regulation of β-catenin by IGFBP2. As described above, over expression of IGFBP2 in the knockdown clones increased β-catenin expression and in the presence of IGF1R inhibitor or FAK inhibitor, IGFBP2 induced β-catenin expression was abolished (Figure 4). Similar results were obtained using MDA-MB-231 cells which lack endogenous IGFBP2 expression (Additional file 7: Figure S3). These results suggest that IGFBP2 regulates β-catenin expression in an IGF1R and integrin dependent manner.

### IGFBP2 and β-catenin staining together correlates with the lymph node metastasis in human breast cancer

**Figure 5:**
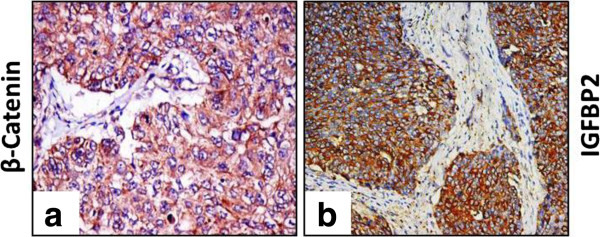
**β-catenin and IGFBP2 expression in breast cancer tissues.** Representative micrographs showing β-catenin staining in cancer tissues. **a** β-catenin staining of a section of IDC 3 breast tumors showing cytoplasmic and membrane staining. **b** represents breast cancer tissue section stained for IGFBP2 showing predominantly cytoplasmic staining.

Since the previous results showed an increase in β-catenin expression upon IGFBP2 over expression, we sought to examine the correlation of β-catenin and IGFBP2 staining in human breast cancer tissues. Towards this we performed IHC on 38 grade III Invasive Ductal Carcinoma tissues for β-catenin and IGFBP2 expression. A representative staining pattern of IGFBP2 and β-catenin expression is depicted in Figure 5. It was observed that 27 out of 38 tumors stained positive for IGFBP2. There was a positive correlation between IGFBP2 and β-catenin expression with 26 out of 27 IGFBP2 positive tumor samples also staining positive for β-catenin (Table 6). Tissues with β-catenin expression exhibited a heterogeneous mixture of membranous and cytosolic β-catenin accumulation. In addition, more lymph node metastasis was observed in patients positive for both IGFBP2 and β-catenin proteins (18/24, 75%) compared with patients with low levels of both proteins (1/24, 4%) (p = 0.0006).

**Table 6:** IGFBP2 and β-catenin expression in breast cancer tissues

***S. No.***	***Case No.***	***IGFBP2***	***β-catenin***	***Nodal status***
**1**	160	+	+	+
**2**	159	+	+	-
**3**	157	+	+	-
**4**	171	+	+	+
**5**	150	+	-	+
**6**	151	-	+	+
**7**	180	+	+	-
**8**	176	+	+	+
**9**	174	+	+	+
**10**	168	+	+	+
**11**	165	+	+	+
**12**	162	+	+	-
**13**	149	+	+	+
**14**	148	+	+	+
**15**	143	+	+	+
**16**	142	+	+	+
**17**	140	+	+	+
**18**	138	+	+	-
**19**	137	+	+	-
**20**	134	+	+	+
**21**	133	+	+	-
**22**	132	+	+	-
**23**	130	+	+	+
**24**	70	+	+	+
**25**	94	-	-	-
**26**	87	+	+	+
**27**	49	+	+	+
**28**	63	+	+	+
**29**	117	+	+	+
**30**	108	-	+	+
**31**	96	-	-	-
**32**	85	-	+	-
**33**	77	-	-	+
**34**	48	-	+	+
**35**	57	-	+	+
**36**	78	-	+	+
**37**	30	-	-	-
**38**	31	-	-	-

No significant association of combined expression of IGFBP2 and β-catenin was observed with ER, PR, Her2 or triple negative receptor status of breast tumors.

## Discussion

Enhanced expression of IGFBP2 is associated with a large number of malignant cancers that include tumors of breast, ovarian, glioma and prostate. Primarily known for its growth inhibitory actions in physiological context, IGFBP2 has now been shown to promote growth and tumorigenesis in numerous cancer cells such as glioma, prostate and colon cancers [15-18]. To gain further insights into the role of IGFBP2 in breast cancer, we have attempted to identify the molecular players in IGFBP2 associated tumorigenesis in breast cancer. To elucidate the molecular targets of IGFBP2, we perturbed IGFBP2 expression by shRNA and the differential gene expression was determined using whole genome microarrays. IGFBP2 knockdown resulted in significant changes in the expression of genes associated with cellular proliferation and tumorigenicity. The down regulated genes were found to be associated with several pathways, notably Cell cycle, p53 and Wnt pathways as revealed by GSEA. Comparison of our data with a previous microarray study of IGFBP2 regulated genes in glioma cells [29] revealed an overlap of about 22% genes with wild type IGFBP2 over expressing cells and 23% genes with RGE mutant IGFBP2 over expressing cells. Pathway comparisons revealed Cell cycle, p53 signaling, oxidative phosphorylation, nucleotide metabolism and Wnt signaling pathway to be common among the two data sets (Additional file 8: Figure S4). To further validate these results in breast cancer tissues, we performed whole genome expression analysis in 19 breast tumors which were categorized as IGFBP2 positive or negative based on immunohistochemical staining pattern. Compared to IGFBP2 negative tumors, IGFBP2 positive tumors showed increased expression of genes belonging to MAPK signaling, Focal adhesion and Wnt signaling.

IGFBP2 correlation with proliferation has been studied extensively in several tumor cells including in breast cancer cells. The effect of IGFBP2 on proliferation has been shown to be context dependent. In prostate, ovarian, nephroblastoma cells, it has a pro proliferative action [19,21-24]. In contrast IGFBP2 has an antiproliferative effect on HEK, Hs578T [32,33]. Our data on the regulation of different pathways such as MAPK, Cell cycle, Focal adhesion and Wnt corroborate the reported functional significance of IGFBP2 with respect to its pro proliferative and tumor promoting roles in breast cancer cells.

One of the important and novel findings from this study is the regulation of Wnt signaling pathway genes by IGFBP2. So far, only IGFBP4 has been reported to activate Wnt signaling pathway in renal cell carcinoma [34]. Activation of canonical Wnt signaling promotes tumorigenesis by regulating cell survival, proliferation and invasion of many cancers [35]. In numerous tumors cytoplasmic and/or nuclear accumulation of β-catenin has been shown to be a strong indicator of aberrant Wnt pathway activation. Elevated cytosolic and nuclear accumulation of β-catenin has been associated with a variety of malignancies and inversely correlated with patient survival [36-39], Wnt activation leads to stabilization and translocation of β-catenin from cytoplasm to the nucleus where it associates with T-cell factor (TCF)/lymphocyte enhancer transcription (LEF) factors to activate target genes that are involved in cell survival, proliferation, and invasion [40,41]. In order to establish Wnt pathway activation by IGFBP2, we examined the canonical Wnt signaling target, β-catenin in IGFBP2 knockdown breast cancer cells. Compared to Vector transfected cells, IGFBP2 knockdown cells showed remarkably decreased levels of β-catenin. When IGFBP2 was re expressed in the knockdown cells, as expected there was substantial increase in β-catenin levels indicating that IGFBP2 regulates β-catenin. Interestingly, inhibition of IGF1R or integrin signaling resulted in the loss of β-catenin regulation by IGFBP2. These data suggest that IGFBP2 acts through IGF1R and integrin pathways in the regulation of β-catenin. Although the mechanisms are not clear, recently Uzoh et al. demonstrated an increased proliferation of prostate cancer cells by IGFBP2 in an IGF1R dependent manner [20]. It is also known that IGF independent actions of IGFBP2 are mediated by the activation of integrin signaling through RGD motif present in the C-terminal region of IGFBP2 protein [30]. Role of integrin receptors in pro-tumorigenic action of tumor cells is well established [42,43]. Hence, it is conceivable that activation of integrin signaling by IGFBP2 leading to FAK phosphorylation may be an important step in the activation of IGF1R by IGFBP2. In congruence with this, it has been reported that activated FAK phosphorylates and stabilizes IGF1R in mouse embryonic fibroblast [44]. Very recently, IGFBP2 in association with IGF1 was found to activate IGF1R in endothelial cells [45]. Taken together, regulation of Wnt pathway by IGFBP2 involves FAK and IGF1R in breast carcinogenesis. However, the mechanism (s) by which FAK and IGF1R signaling converge on the regulation of Wnt pathway by IGFBP2 needs further investigations.

Another important finding from our data is the correlation of IGFBP2 over expression with elevated β-catenin levels in breast tumors. In humans, breast tumors frequently exhibit elevated levels of IGFBP2 [12] and β-catenin, with higher expression levels of β-catenin correlating with a decreased patient survival [39]. In mice, over expression of an activated β-catenin leads to the development of mammary hyperplasia and adenocarcinomas [46]. These studies coupled with our data suggest that regulation of β-catenin could be an important step for the pro-tumorigenic actions of IGFBP2. Most significantly, when both IGFBP2 and β-catenin expression was correlated with the lymph node status of breast cancers, we found a significant association of IGFBP2 and β-catenin staining with increased lymph node metastasis in comparison with tumors which did not show staining for either protein. Interestingly, in a previous report, expression of IGFBP2 and IGFBP5 were correlated with increased lymph node metastasis in T1 breast carcinoma. However our data shows a significant positive correlation of IGFBP2 and β-catenin in lymph node metastasis. Hence, evaluation of IGFBP2, IGFBP5 along with β-catenin may provide a stronger predictive value for the prognosis of breast cancer.

## Conclusion

This study highlights the pathways and genes regulated by IGFBP2 in breast cancer. Most importantly, this study reports regulation of β-catenin by IGFBP2 and their association in the lymph node metastasis. These findings are highly relevant in the prediction of breast cancer progression.

## Methods

All the tissues for this study were collected after obtaining written informed consent from the patients. This study and the protocols were approved by the Institutional Ethics Committee of Kidwai Memorial Institute of Oncology, where the patients were treated.

### Cell culture and transfection

BT474, a breast cancer cell-line was cultured in DMEM (Sigma-Aldrich, USA) with 10% foetal bovine serum (FBS), 100 units/ml penicillin and 100 μg/ml streptomycin, 2.5 μg/ml fungizone (Invitrogen Life Sciences, USA). All the cells were maintained at 37°C in a humid atmosphere with 5% CO_2_. Transfections were performed using Lipofectamine 2000 (Invitrogen) based on the manufacturer’s instructions. In brief, breast cancer cells were transfected with IGFBP2 shRNA expression vector (Origene, Cat no. TR316590) or empty vector (Origene, Cat no. TR20003) and 48 hrs after transfection puromycin (1 μg/ml, Calbiochem) was added to the growth medium. Selection medium was replaced every 2–3 days until individual clones could be identified. After 3 weeks of selection, fourteen puromycin resistant clones of BT474 cells were isolated and expanded in the selective medium. Two clones (C5 and C12) which showed significant down regulation of IGFBP2 expression were selected for further experiments Reversion of IGFBP2 expression in IGFBP2 knockdown cells was achieved by transfecting IGFBP2 cDNA sub cloned into pcDNA3.1 vector (Invitrogen). Pathway inhibitor treatments were performed using IGF1R inhibitor (PPP, 10 μM, Calbiochem Cat. No. 407247) and Focal Adhesion Kinase inhibitor (PP2, 10 μM, Calbiochem Cat. No. 529573).

### Immunoblot analysis

For immunoblot analysis, cells were grown in growth medium till they achieved 50-70% confluency, washed with serum free DMEM and cultured in serum free medium for another 48 h. The spent medium was collected, concentrated using centrifugal filter units (Millipore, Amicon ultra-3 k) and equal amounts of protein as determined by the Bio-Rad DC protein assay (Bio-Rad, USA) were separated on 12.5-15% polyacrylamide gel and electrophoretically transferred onto PVDF membranes (Immobilin P, Millipore). Membranes were pre-incubated for 1 h with 5% non-fat dry milk (Fluka, Sigma-Aldrich) in Tris buffered-saline containing 0.1% Tween 20 (TBST) and then were incubated overnight with primary antibody. (IGFBP2 C-18: sc-6001, Santa Cruz Biotechnology, Inc, CA). Membranes were washed thrice for 15 min in TBST at room temperature, incubated with appropriate horseradish-peroxidase conjugated IgG (Sigma-Aldrich) at a dilution of 1:2000 for 1 h at room temperature and the complex detected using Super Signal West Femto chemiluminescence (Pierce, Thermo Scientific), as per the manufacturer’s instructions.

### RNA extraction and gene expression profiling

Total RNA from frozen tumor tissues and tumor cells was extracted using the TRI reagent (Sigma-Aldrich) according to the manufacturer’s protocol. The concentration of RNA was estimated by measuring the absorbance at 260 nm (Nano Drop ND-1000 spectrophotometer) and integrity was verified on a denaturing 1% MOPS-formaldehyde agarose gel followed by ethidium bromide staining. For expression profiling, microarray experiments using whole genome human arrays (4×44K, Agilent) were used. The microarray hybridizations were performed as described before [47]. Microarray analysis was performed by R-Bioconductor (limma package) using subtract method for background correction [48]. Loess normalization was applied for dye bias and Quantile normalization was applied for spatial variation [49]. Linear model and empirical Bayes methods (limma) was used for assessing differentially regulated genes [50]. Benjamini Hochberg correction was applied for P value correction. Hierarchical cluster was done by Mev4.1 using Euclidean distance metric. The data was clustered by averaged linkage [51]. Adjusted p value cut-off was used as 0.05 for differentially regulated genes. Gene expression data are deposited into GEO (Clone arrays: GSE40682, Breast cancer tissue arrays: GSE40206).

### Real-time qPCR assay

For RT-PCR, cDNA was synthesised from total RNA using the cDNA Archive kit (Applied Bio systems, USA). cDNA equivalent to 10 ng of total RNA was used for all the PCR reactions using Dynamo SYBR green mix (Finnzymes, Finland) in ABI Prism 7900HT sequence detection system (Applied Bio systems, USA). The sequences of the primers are shown in Additional file 9: Table S5. The analysis has been done using SDS 2.1 software (Applied Bio systems, USA). For normalization of RT-PCR data, ribosomal protein L35a (RPL 35a) and TATA Binding Protein (TBP) were used for cells and tissues, respectively.

### Immunoflourescence

Cells were grown on sterile cover-slips till they were about 50% confluent. The growth medium was discarded; cells were washed twice with chilled DPBS and were fixed in ice cold methanol for 10 minutes at −20°C. The fixed cells were then washed with DPBS thrice. For blocking non-specific binding of the antibodies, the cells were incubated with 1% BSA in PBS for 60 min followed by overnight incubation with protein specific antibodies (β-catenin, 1:50; IGFBP2, 1:25) in a humidified chamber at 4°C. After the overnight incubation, the cells were washed thrice with PBS and incubated with the secondary antibody, 1:1500 dilution of alexa flur 488 (anti-rabbit, for β-catenin) and alexa flur 633 (anti-goat for IGFBP2) (Molecular probes, Invitrogen, USA) in PBS for 1 hour in dark. All steps thereafter were performed in the dark. After 1 h, the cells were again washed thrice with PBS and counterstained with 33 μg/ml Propidium Iodide for 5 minutes and mounted in anti‒fade solution on clean slides. The stained cells were visualized using a confocal microscope (LSM 510 Meta, Carl-Zeiss) and were photographed.

### Tissue samples and immunohistochemistry

For histology, sections of breast tumor tissues were obtained from blocks archived in the Department of Pathology at the Kidwai Memorial Institute of Oncology (KMIO). The status of estrogen receptor (ER), progesterone receptor (PR), Her2/neu, and pathological data like tumor grade, size and lymph node status were obtained from the pathology records of the respective patients. Tissue sections (5 μm) from the paraffin embedded tumor specimens were collected on silane-coated slides and immunohistochemistry for IGFBP2 and β-catenin was performed on 38 samples. Antigen retrieval was done by heat treatment of the deparaffinised sections in Citrate buffer (10 mM; pH 6.0). After the initial processing steps, sections were incubated overnight with respective primary antibodies - IGFBP2 (C-18: sc-6001, Santa Cruz Biotechnology, Inc, CA) and β-catenin (C 2206, Sigma-Aldrich), at 4°C. This was followed by incubation with the linked streptavidin- biotinylated secondary antibody (Universal LSAB, DAKO, Denmark) for IGFBP2 and with supersensitive non-biotin horseradish peroxidase detection system (QD440-XAK, Biogenex) for β-catenin antibodies. 3, 3’-Diaminobenzidine (Sigma-Aldrich) was used as the chromogenic substrate.

### Evaluation of immunohistochemistry

The scoring method used for IGFBP2 and β-catenin expression was based on semi quantitative scoring method as described before [52] where both intensity and percentage of cells with positive staining were counted and a combined score was given. The combined score was arrived by the multiplication product of both the scores. The scores are, (1) percentage of cells: no staining = 0; 10% or less of cells stained = 1; 11–50% of cells stained = 2; and 50% or more of cells stained =3; (2) intensity: no staining = 0, weak staining = 1, moderate staining = 2, and strong staining = 3. Thus, the combined scores ranged from 0–9. Only scores from 4–9 were considered positive for staining.

#### Statistical analysis

Statistical significance for all experimental analyses (except microarray) was determined by Student’s t-test or one-way analysis of variance GraphPad Prism 5.0 software (GraphPad Software, Inc., La Jolla, CA, USA). For correlation analysis Fisher’s exact test was utilized.

## Competing interests

The authors declare that they have no competing interests.

## Authors’ contributions

PS planned, executed, interpreted experiments, manuscript writing; VRP, SP and AB executed some experiments; NK did the microarray analysis; MVK and GM contributed in the planning and recruitment of patients and samples, clinical evaluation; PK, planned the experiments; manuscript writing and resources. All authors read and approved the final manuscript.

## Supplementary Material

Additional file 1 List of common probe between IGFBP2 Knockdown clones C5 and C12. Click here for file

Additional file 2: Figure S1 Gene expression changes in IGFBP2 knockdown cells upon IGFBP2 over expression. Cells were plated and 24 h later transfected with pcDNA3.1-IGFBP2 and /or pcDNA3.1 vector. 48 h post transfection, RNA was extracted and gene expression was analyzed by Semi quantitative RT-PCR analysis. Representative ethidium bromide gel shows the expression of genes regulated upon forced expression of IGFBP2 in **a**) clone C12 and **b**) clone C5. Expression values were quantitated and the graph (right) represents fold change over control after normalization with the expression of RPL35A. Click here for file

Additional file 3 List of Differentially expressed probes between IGFBP2 positive and negative breast tumors. Click here for file

Additional file 4: Figure S2 Validation of Wnt target genes in IGFBP2 positive and IGFBP2 negative tumors. Scatter plots of differentially regulated genes in tumor tissues compared to the expression in normal tissues. Log 2-transformed gene expression ratios obtained from real-time quantitative PCR analysis normalized to TBP expression are plotted. Each dot represents data derived from one sample. Click here for file

Additional file 5 List of differentially regulated probes common between IGFBP2 knockdown clones and IGFBP2 positive/negative tumors. Click here for file

Additional file 6 List of pathways associated with differentially regulated genes common between IGFBP2 knockdown clones and IGFBP2 positive/negative tumors. Click here for file

Additional file 7: Figure S3 Regulation of β-catenin by IGFBP2 is IGF1R and FAK dependent. MDA-MB-231 cells were transfected with IGFBP2 and 36 h. after transfection cells were fixed and analyzed for β-catenin and IGFBP2 protein. For inhibitor treatements, 24 h. after transfection cells were treated with IGF1R or FAK inhibitor for 12 h. Cells were fixed and analyzed for β-catenin and IGFBP2 expression. Expression of β-catenin and IGFBP2 is shown in green and blue, respectively. Nucleus was stained using propidium iodide (PI) as shown in red Original magnification was 63×. V, Vector control; OE, Over expression; Inh, inhibitor. Click here for file

Additional file 8: Figure S4 Comparison of IGFBP2 regulated genes in knock down cells with other available data sets. **a**) Venn diagram showing genes common between IGFBP2 over expressing glioma cells (GEO accession no. GSE35467) and IGFBP2 knock down breast cancer cells (Additional file 1: Table S1). **b**) Venn diagram showing genes common between RGE mutant IGFBP2 over expressing glioma cells (GEO accession no. GSE35467) and IGFBP2 knock down breast cancer cells (Table S1). OV, over expression; KD, knockdown; RGE, RGE mutant IGFBP2. Click here for file

Additional file 9 List of primer sequences used for qPCR. Click here for file
