# Supplementary figures and images for: Regulation of protumorigenic pathways by Insulin like growth factor binding protein2 and its association along with β-catenin in breast cancer lymph node metastasis
Source: Mol Cancer. 2013 Jun 16;12:63. doi: 10.1186/1476-4598-12-63 (PMC3698021; doi:10.1186/1476-4598-12-63)

## Slide 1
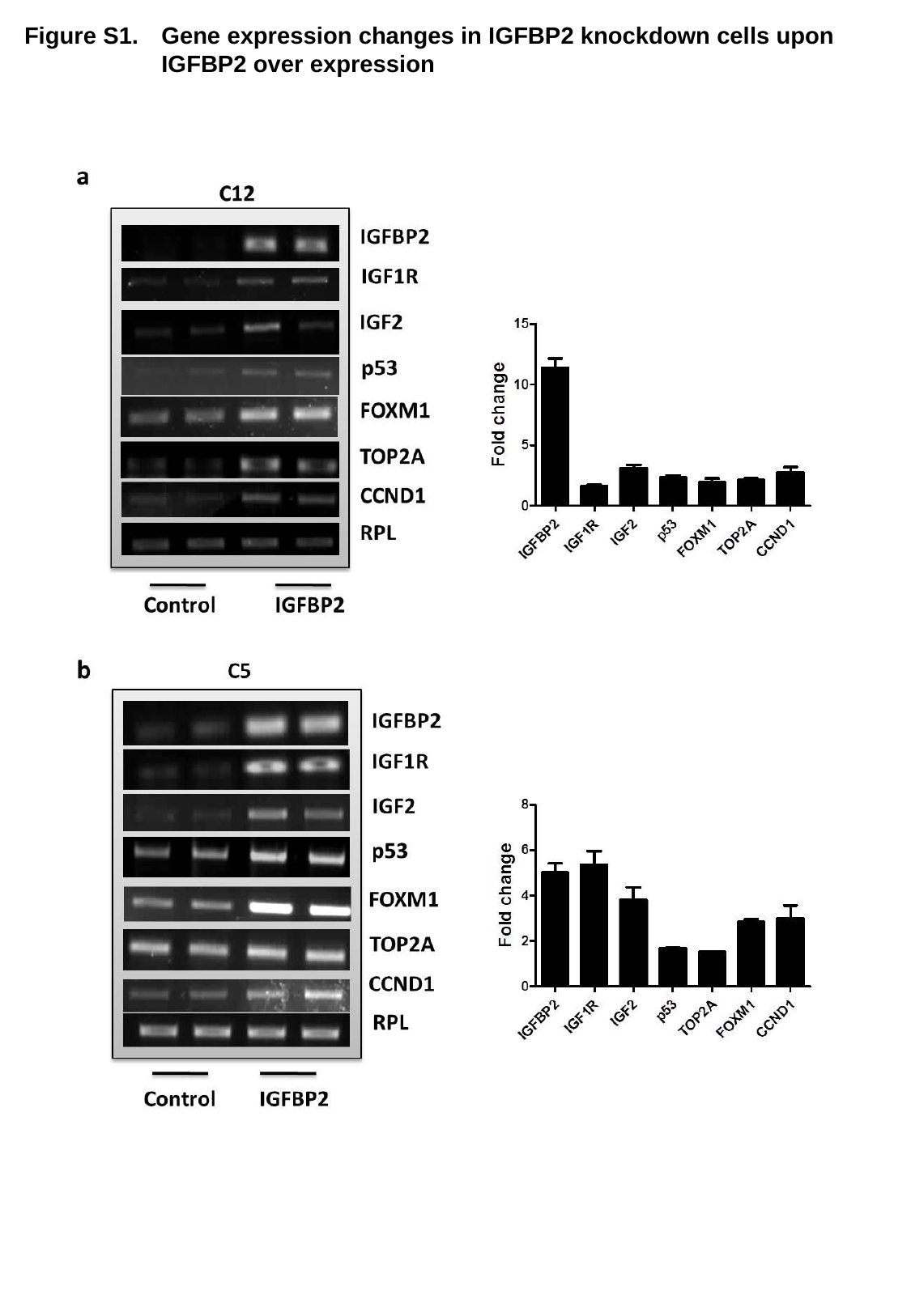

Figure S1.
Gene expression changes in IGFBP2 knockdown cells upon IGFBP2 over expression

Supplement: Additional file 2: Figure S1 — Gene expression changes in IGFBP2 knockdown cells upon IGFBP2 over expression. Cells were plated and 24 h later transfected with pcDNA3.1-IGFBP2 and /or pcDNA3.1 vector. 48 h post transfection, RNA was extracted and gene expression was analyzed by Semi quantitative RT-PCR analysis. Representative ethidium bromide gel shows the expression of genes regulated upon forced expression of IGFBP2 in a) clone C12 and b) clone C5. Expression values were quantitated and the graph (right) represents fold change over control after normalization with the expression of RPL35A. [file 1476-4598-12-63-S2.ppt]

## Slide 1
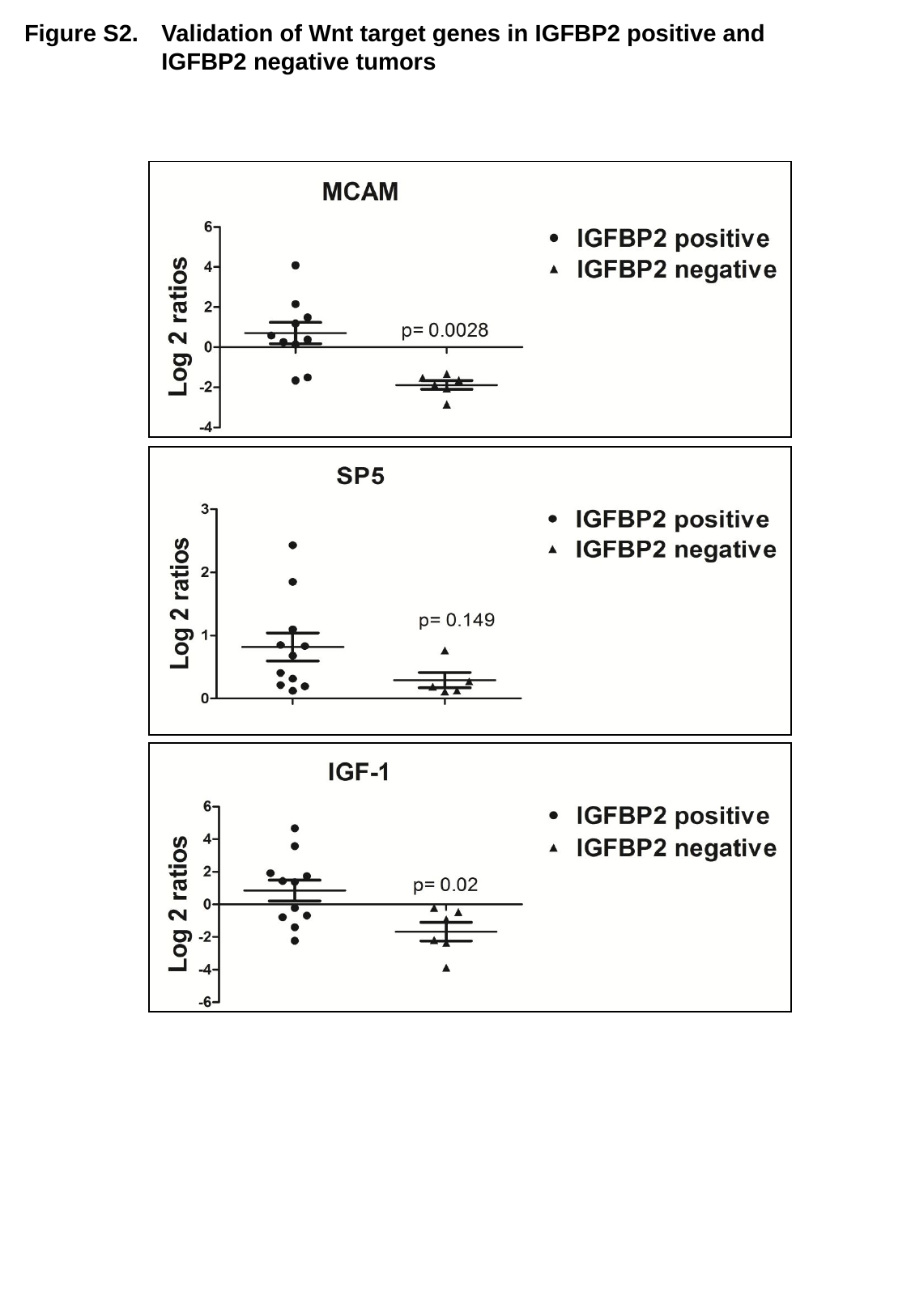

Figure S2.
Validation of Wnt target genes in IGFBP2 positive and IGFBP2 negative tumors

Supplement: Additional file 4: Figure S2 — Validation of Wnt target genes in IGFBP2 positive and IGFBP2 negative tumors. Scatter plots of differentially regulated genes in tumor tissues compared to the expression in normal tissues. Log 2-transformed gene expression ratios obtained from real-time quantitative PCR analysis normalized to TBP expression are plotted. Each dot represents data derived from one sample. [file 1476-4598-12-63-S4.ppt]

## Slide 1
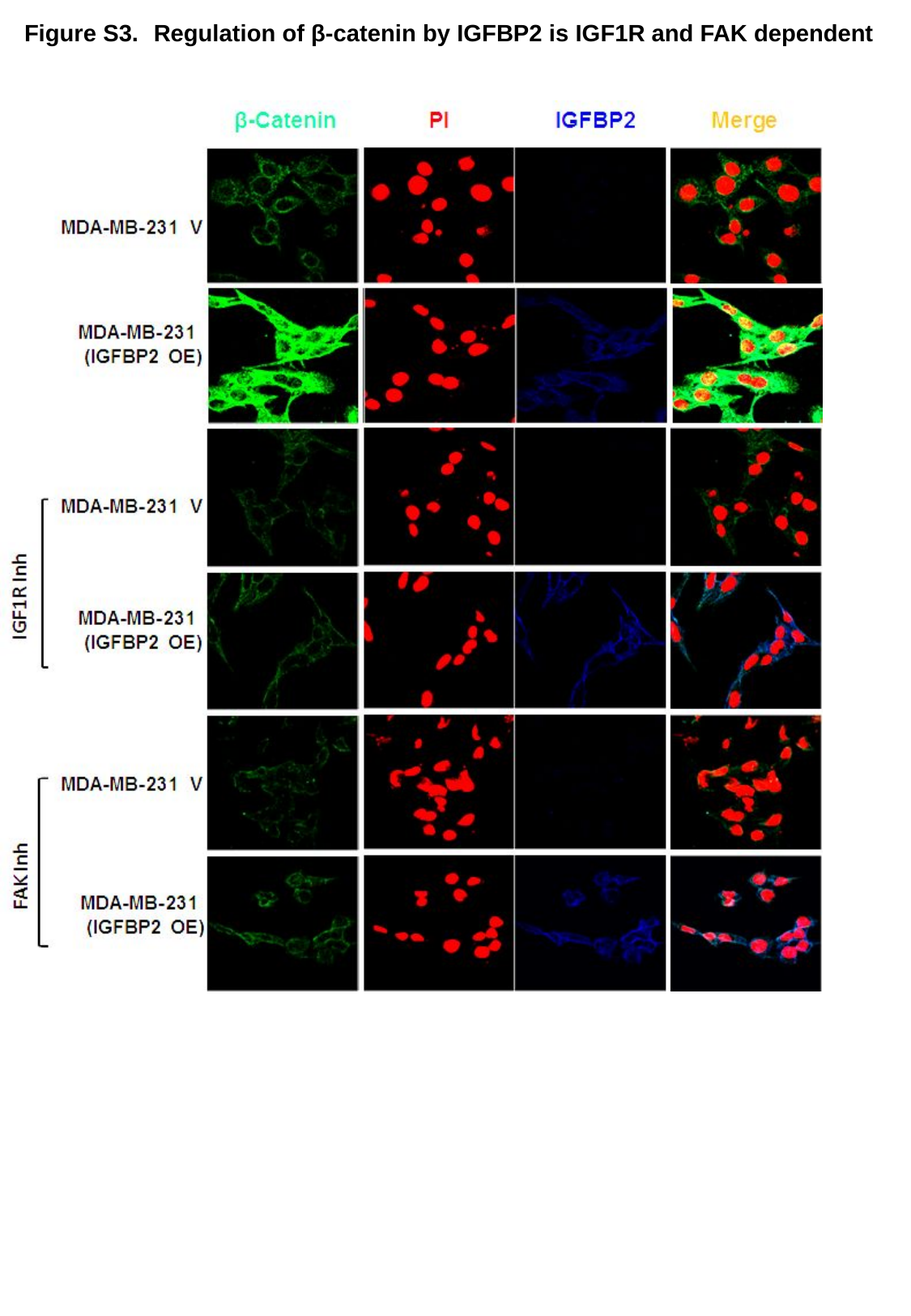

Figure S3.
Regulation of β-catenin by IGFBP2 is IGF1R and FAK dependent

Supplement: Additional file 7: Figure S3 — Regulation of β-catenin by IGFBP2 is IGF1R and FAK dependent. MDA-MB-231 cells were transfected with IGFBP2 and 36 h. after transfection cells were fixed and analyzed for β-catenin and IGFBP2 protein. For inhibitor treatements, 24 h. after transfection cells were treated with IGF1R or FAK inhibitor for 12 h. Cells were fixed and analyzed for β-catenin and IGFBP2 expression. Expression of β-catenin and IGFBP2 is shown in green and blue, respectively. Nucleus was stained using propidium iodide (PI) as shown in red Original magnification was 63×. V, Vector control; OE, Over expression; Inh, inhibitor. [file 1476-4598-12-63-S7.ppt]
