# Supplementary material for: Regulation of protumorigenic pathways by Insulin like growth factor binding protein2 and its association along with β-catenin in breast cancer lymph node metastasis
Source: Mol Cancer. 2013 Jun 16;12:63. doi: 10.1186/1476-4598-12-63 (PMC3698021; doi:10.1186/1476-4598-12-63)
Supplement: Additional file 8: Figure S4 — Comparison of IGFBP2 regulated genes in knock down cells with other available data sets. a) Venn diagram showing genes common between IGFBP2 over expressing glioma cells (GEO accession no. GSE35467) and IGFBP2 knock down breast cancer cells (Additional file 1: Table S1). b) Venn diagram showing genes common between RGE mutant IGFBP2 over expressing glioma cells (GEO accession no. GSE35467) and IGFBP2 knock down breast cancer cells (Table S1). OV, over expression; KD, knockdown; RGE, RGE mutant IGFBP2. [file 1476-4598-12-63-S8.ppt]

## Slide 1
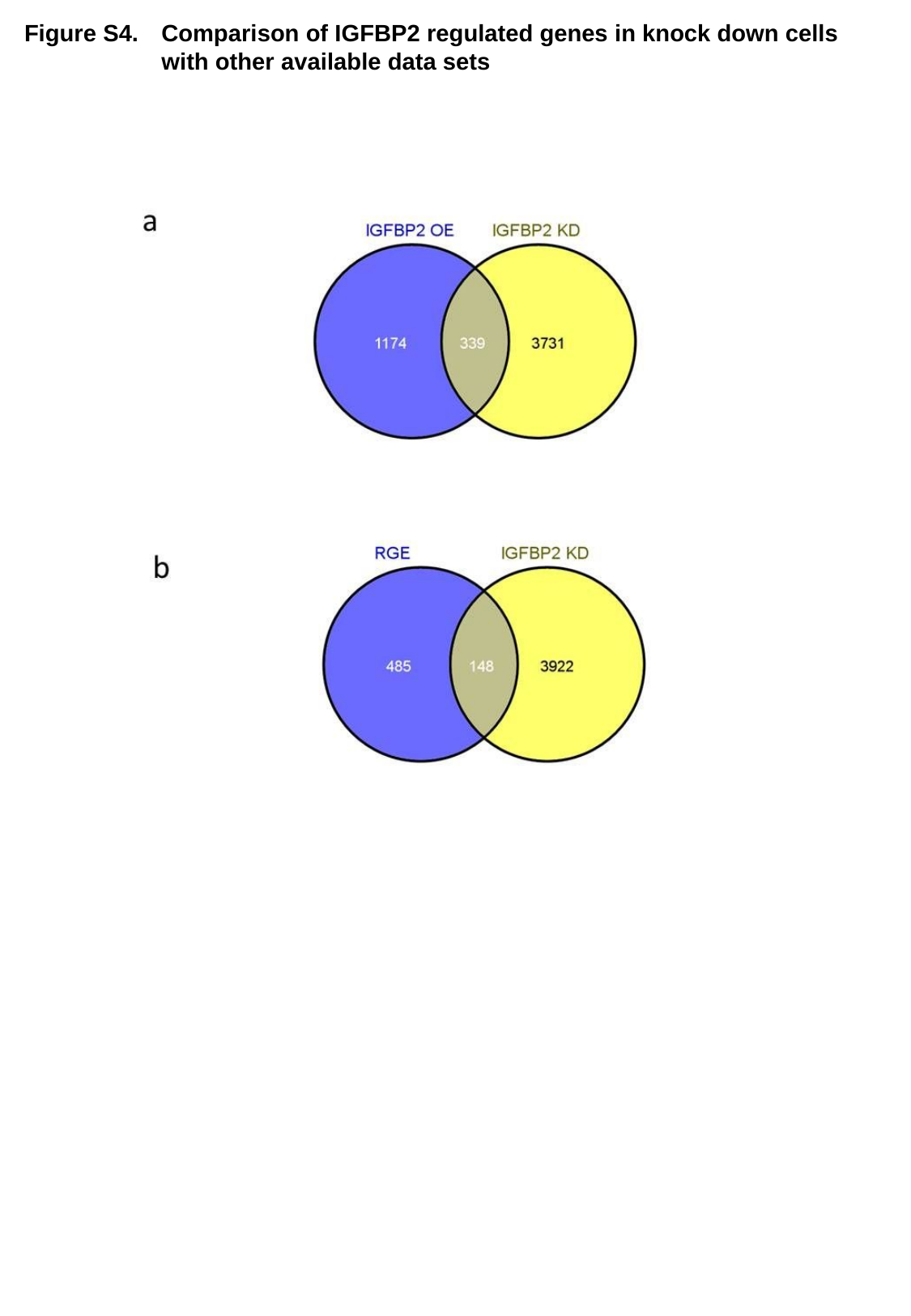

Figure S4.
Comparison of IGFBP2 regulated genes in knock down cells with other available data sets
